# Chiral Speciation in Terrestrial Pulmonate Snails

**DOI:** 10.1371/journal.pone.0034005

**Published:** 2012-04-20

**Authors:** Edmund Gittenberger, Thomas D. Hamann, Takahiro Asami

**Affiliations:** 1 Netherlands Centre for Biodiversity, Leiden, The Netherlands; 2 Department of Biology, Shinshu University, Matsumoto, Japan; Brigham Young University, United States of America

## Abstract

On the basis of data in the literature, the percentages of dextral versus sinistral species of snails have been calculated for western Europe, Turkey, North America (north of Mexico), and Japan. When the family of Clausiliidae is represented, about a quarter of all snail species may be sinistral, whereas less than one per cent of the species may be sinistral where that family does not occur. The number of single-gene speciation events on the basis of chirality, resulting in the origin of mirror image species, is not closely linked to the percentage of sinistral versus dextral species in a particular region. Turkey is nevertheless exceptional by both a high percentage of sinistral species and a high number of speciation events resulting in mirror image species. Shell morphology and genetic background may influence the ease of chirality-linked speciation, whereas sinistrality may additionally be selected against by internal selection. For the Clausiliidae, the fossil record and the recent fauna suggest that successful reversals in coiling direction occurred with a frequency of once every three to four million years.

## Introduction

Metazoan animals generally exhibit left-right asymmetry (chirality) in their internal structure, although many of them are externally symmetric in basic body plans. Development of this chirality can be reversed by mutation [1], [2], [3]. This results in left-right reversal in whole-body structure. In most animal groups however, few species that are fixed for reversal throughout development and thus reversed in visceral chirality, are found [4], [5]. Considering this generality, clockwise-coiled (dextral) and counterclockwise-coiled (sinistral) gastropods represent unique examples of the evolution of species that are reversed in whole-body chirality including coiling direction as well as visceral bilateral asymmetry [2]. In some groups, coiling direction is secondarily reversed and thus does not match with the polarity of visceral asymmetry [3].

**Figure 1:**
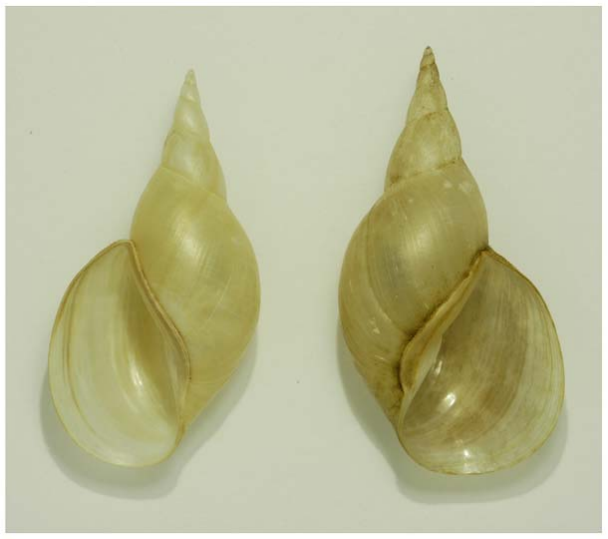
A sinistral mutant (left) and a dextral (right) wild-type shell of *Lymnaea stagnalis* from artificial populations at Leiden University.

Most snails' shells are dextral, versus a minority that is sinistral (Fig. 1). With few exceptions (e.g., [6], [7]), exact numbers or percentages of the two alternatives are hardly ever mentioned, neither for snails in general nor for species in a particular geographical region [8]–[20]. Apparently, statements on the rarity of sinistral taxa are mainly based on guesswork, not on real counting and comparing of dextral and sinistral species in a particular fauna let alone worldwide. In this study we counted the numbers of sinistral and dextral species recorded in the literature on some relatively well-known, terrestrial, pulmonate faunas, to investigate regional differences and the frequency of the origin of opposite whole body chirality (chiral speciation).

Despite some misleading contrary statements in the literature (e.g., [20], [21]), very few species are really dimorphic for the polarity of whole-body chirality, with mixed populations of dextral and sinistral individuals occurring at high percentages. *Lymnaea* ( = *Radix*) *peregra* and *Lymnaea stagnalis* for example, which are mentioned by these authors, cannot be called dimorphic for chiral polarity, since in both species sinistral specimens are extremely rare in nature [5], [22]. Apparently not all authors realise that “cases of exceptional individuals with reverse chirality are known in many gastropods” [23]. Only in captivity, large sinistral populations of *L. stagnalis* occur these days, all of which descending from some mutants found in a small pond along the Danube in southern Germany by Gerhard Falkner and used to get homozygous populations of both the wild type and the mutant at Leiden University by the first author of this article [22].

Only in *Amphidromus* Albers, 1850, are many species with certainty dimorphic with sinistral and dextral specimens occurring in mixed populations [2], [3], [12], [24], [25], [26], [27]. According to the taxonomic literature, some other gastropod species are also dimorphic in chirality. However, in those species the two forms may be more or less clearly distinguishable on the basis of additional characters and may have mutually exclusive ranges, which may be more or less clearly interconnected by a hybrid zone, so that their taxonomic status is to some extent disputable. For example, according to Schütt [28], *Pseudochondrula arctespira* (Mousson, 1874), *P. sebasteana* (Forcart, 1940) *Orculella menkhorsti* Hausdorf, 1996, and *Schileykula trapezensis* (Stojaspal, 1981), all from Turkey, are dimorphic for coiling direction.

From an evolutionary perspective, the percentage of sinistral versus dextral species is less meaningful than the number of chiral speciation events. The rarity of sinistrality in nature suggests that speciation events that are accompanied by a right-left reversal in development are very unusual [29]. In Southeast Asia however, snake predation of dextral snails may have promoted the evolution of sinistral snail species by reversal [30], [31].

For four terrestrial pulmonate faunas, selected on the basis of convenience, i.e. the presence of compiling monographs, the percentage of sinistral species is calculated. These data are then used to derive the number of corresponding events of chiral speciation. Apart from that, the number of established reversals during the evolutionary history of the Clausiliidae is counted. This speciose family has been selected because it can be recognized relatively easily by the slender, usually sinistral shells, with a unique closing apparatus, i.e. a combination of lamellae and the clausilium, which gives the family its name. This undisputedly monophyletic group most probably had its evolutionary origin with a sinistral ancestral species over 65M years ago in the Cretaceous [32], so that dextral taxa indicate independent events of chirality reversal from sinistrality to dextrality. Despite the incompleteness of the fossil record, the data on Clausiliidae may give an idea about the frequency of such events in a family where shell shape and genetic background both facilitate chiral speciation [18], [19], [22], [33].

## Materials and Methods

On the basis of the compiling monographs of Kerney, Cameron and Jungbluth [34], Schütt [28], Pilsbry [35] and Minato [36], [37], the numbers of dextral and sinistral species in western Europe, Turkey, North America (north of Mexico), and Japan were counted. These regions, referred to as Europe, Turkey, North America and Japan in the following text, were selected because of the large numbers of extant species in their molluscan faunas and because useful monographs summarizing the species compositions are available. The data were taken uncritically from the literature, assuming that alternative views on single species delimitations will not seriously influence our general conclusions.

As long as thorough phylogeny reconstructions based on morphological and molecular data are not available, congeneric dextral and sinistral species are considered descendants of a single ancestral species referring to a single reversal event.

While counting dextral versus sinistral species, the few so-called dimorphic species are counted as both dextral and sinistral, irrespective of the rate of gene flow (usually unknown) between sinistral and dextral morphs. For the notes on fossil and recent Clausiliidae, the data provided by Nordsieck [32] were used as a basis.

## Results

### Regional Sinistrality

In Europe, 84.0% of the 331 species of snails are dextral, and 16.0% are sinistral [34]. When the 50 clausiliid species are not counted, 1.1% of the 281 remaining species are sinistral. The sinistral species that are not classified as Clausiliidae belong to two families, viz. Enidae (*Jaminia quadridens* (Müller, 1774)) and Vertiginidae (2 species: *Vertigo (V.) pusilla* Müller, 1774; *V. (Vertilla) angustior* Jeffreys, 1830).

In Turkey, 75.2% of the 541 species of snails are dextral, and 24.8% are sinistral [28]. When the 111 clausiliid species are not counted, 5.3% of the 430 remaining species are sinistral. The sinistral species that do not belong to the Clausiliidae are classified with three families: Orculidae (4 species: *Orculella heterostropha* (O. Boettger, 1905), *O. sinistrorsa* Hausdorf, 1996, *Schileykula inversa* Schütt, 1993, *S. contraria* Neubert, 1993), Vertiginidae (2 species: *Vertigo (V.) pusilla* Müller, 1774; *V. (Vertilla) angustior* Jeffreys, 1830), and Enidae (17 species: *Pseudochondrula arctespira* (Mousson, 1874); *P. armeniaca* (Mortillet, 1854); *P. sebasteana* (Forcart, 1940); *P. florenskii* (Rosen, 1914); *P. seductilis* (Rossmässler, 1837); *P. maden* Schütt, 2005; *Imparietula ridvani* Schütt, 1995; *Ljudmilena armeniaca* (Ancey, 1893); *L. excellens* (Retowski, 1889); *L. tricollis* (Mousson, 1876); *Jaminia loewii* (Philippi, 1844); *Chondrus tournefortianus* (A. Férussac, 1821); *Ramusculus laevitortus* Schütt, 1995; *Thoanteus corneus* Hausdorf, 1993; *T. zilchi* Hausdorf, 1993; *Eubrephulus orientalis* (L. Pfeiffer, 1848); *Multidentula microdon* (Schütt 1995)).

In North America (north of Mexico), 99.4% of the 689 species are dextral and 0.6% are sinistral [35]. One species, i.e. *Holospira roemeri* (L. Pfeiffer, 1848), could be called sinistroid [38]. The sinistral species belong to two families, viz. Gastrocoptidae (*Gastrocopta perversa* (Sterki, 1898)) and Pupillidae (3 species: *Pupilla nefas* Pilsbry & Ferriss, 1910; *P*. *sinistra* Franzen, 1946; *P*. *syngenes* (Pilsbry, 1890)).

For Japan 861 gastropod species are listed, 82.0% of which are dextral and 18.0% are sinistral [36], [37]. When the 149 clausiliid species are not counted, 0.8% of the 712 remaining species are sinistral. The latter group of sinistral species belongs to the Camaenidae Pilsbry, 1895 (*Euhadra decorata* (Pilsbry & Hirase, 1903); *E. echigoensis* Murayama, Takizawa & Habe, 1991; *E. murayamai* Habe, 1976; *E. quaesita* (Deshayes, 1850); *E. scaevola* (Martens, 1877); *Satsuma perversa* Pilsbry, 1931; *Satsuma* spec.).

Only the two sinistral species of Vertiginidae occur in more than one region, i.e. Europe and Turkey. The four regions differ in the families that are represented, in numbers of species, and in percentages of sinistrality within the families (Table 1).

**Table 1:** Numbers and percentages of dextral and sinistral species in the various families, in the four regions.

	Total	Dextral	Sinistral	S−C
**Europe**	331	278 (84.0%)	53 (16.0%)	1.1%
Vertiginidae	22	20 (91%)	2 (9%)	-
Orculidae	5	5 (100%)	-	-
Gastrocoptidae	-	-	-	-
Pupillidae	8	8 (100%)	-	-
Enidae	5	4 (80%)	1 (20%)	
Clausiliidae	50	-	50 (100%)	-
Camaenidae	1	1 (100%)	-	-
**Turkey**	541	407 (75.2%)	134 (24.8%)	5.3%
Vertiginidae	10	8 (80%)	2 (20%)	-
Orculidae	38	34 (90%)	4 (10%)	-
Gastrocoptidae	-	-	-	-
Pupillidae	5	5 (100%)	-	-
Enidae	92	75 (82%)	17 (18%)	-
Clausiliidae	111	-	111 (100%)	-
Camaenidae	-	-	-	-
**N. America**	689	685 (99.4%)	4 (0.6%)	0.6%
Vertiginidae	37	37 (100%)	-	-
Orculidae	-	-	-	-
Gastrocoptidae	25	24 (96%)	1 (4%)	-
Pupillidae	11	8 (73%)	3 (27%)	-
Enidae	-	-	-	-
Clausiliidae	-	-	-	-
Camaenidae	-	-	-	-
**Japan**	861	706 (82%)	155 (18%)	0.8%
Vertiginidae	8	8 (100%)	-	-
Orculidae	-	-	-	-
Gastrocoptidae	-	-	-	-
Pupillidae	2	2 (100%)	-	-
Enidae	16	16 (100%)	-	-
Clausiliidae	149	-	149 (100%)	-
Camaenidae	196	190 (97%)	6 (3%)	-

Only the sinistral camaenid species have shells that are not clearly higher than broad. S−C = percentage of sinistrality when clausiliid species are excluded. We follow Wade et al. [39] in considering Bradybaenidae Pilsbry, 1934, a junior synonym of Camaenidae Pilsbry, 1895.

### Chirality among the Clausiliidae

Both the minimum number of reversals and the number of such events according to Nordsieck [32], are indicated in an additive way between brackets after the names of the taxa. The numbers differ because as long as modern phylogeny reconstructions are lacking, we consider groups of congeneric dextral clausiliid species as clades, referring to a single case of chiral speciation. Among the fossil Phaedusinae, only *Disjunctaria* (reversal 1) is dextral. The poorly known nominal genus *Cirrobasis* Conrad, 1874, was provisionally classified with the Clausiliidae, Neniinae, by Zilch [40], but is not accepted as a clausiliid genus by Nordsieck (personal communication, 2010).

For the recent Phaedusinae, 6 to 10 reversals to dextrality are documented in *Synprosphyma (Excussispira)* Lindholm, 1925 (2, 3), with 3 species, *Sinigena* Lindholm, 1925 (3, 4), with 2 species; *Oospira (Oospira)* Blanford, 1872 (4, 7), with 6 species; *Oospira (Formosana)* O. Boettger, 1877 (5, 9), with 10 species; *Oospira (Leptacme) sykesi* (Bavay & Dautzenberg, 1899) (6, 10); *Streptodera trachelostropha* (Moellendorff, 1885) (7, 11). Among the Serrulininae only *Tsoukatosia* Gittenberger, 2000 (8, 12), with at least 3 species, is dextral. For the Neniinae only a single dextral species is known, viz. *Incaglaia dextroversa* (Pilsbry, 1949) (9, 13). The Alopiinae are richer in dextral taxa with: *Alopia* H. & A. Adams, 1855 (10, 14+) (problematic, see the discussion); *Albinaria* Vest, 1867 (11, 16+), with 4 species; *Sericata dextrorsa* (O. Boettger, 1877) (12, 17+); *Cristataria colbeauiana* (L. Pfeiffer, 1861) (13, ?18+); *Leucostigma convertita* (Flach, 1907) (14, 20+). This implies a number of at least 14 and maybe over 20 reversals within the family of Clausiliidae. For the other subfamilies, viz. Laminiferinae, Garnieriinae and Clausiliinae, no dextral taxa are known.

### Shell Shape and Chirality

All the sinistral species in North America, Europe and Turkey have shells that are much higher than broad. In the research areas, sinistral species with a shell that is broader than high, are only known from Japan, viz. the *Euhadra* and *Satsuma* species.

## Discussion

### Limitations

With only two alternatives, i.e. dextral versus sinistral, iterative reversals cannot be recognized. Starting from the usual assumption that in general dextrality is the plesiomorph condition among the gastropods, a dextral clausiliid species refers to minimally two reversals, i.e. a primary one resulting in the ancestral species of the family and a secondary one for the reversal to dextrality. Only the latter reversal has been counted here. In other families, dextral taxa may also result from either an even number of reversals in chirality, or none, as shown in *Euhadra* [41]. As a consequence, the real number of reversals in coiling will never be known, unless phylogeny reconstructions become available. The fossil record for non-marine gastropods is too incomplete to solve this problem.

### Regional Differences in Chirality

There are extreme differences in the number and percentage of sinistral versus dextral gastropod species among various recent molluscan faunas. This varies from 0.6% of 689 for North America to 16.0% of 331 in Europe, 18.0% of 860 in Japan, and 24.8% of 532 species reported from Turkey. These numbers largely depend on the presence or absence of only a single, but speciose, gastropod family, viz. the Clausiliidae, which is not represented in North America. For that reason North America stands apart with only 0.6% sinistrality among its terrestrial gastropod species. When the Clausiliidae are left out, the percentages of sinistral species for the four regions are 0.6, 1.1, 0.8, and 5.3 %, respectively. In that case Turkey exhibits an exceptionally high proportion, linked to the presence of the relatively speciose families Orculidae and Enidae.

The recent European molluscan fauna bears witness to four reversals, which occurred in three families, viz. Clausiliidae (1 reversal), Vertiginidae (2) and Enidae (1). For North America two reversals can be recognized, which occurred in two families, viz. Gastrocoptidae and Pupillidae. For Japan three reversals are recorded, for Clausiliidae and Camaenidae, respectively. With two reversals that are documented, North America does not differ significantly from Europe and Japan, with four and three, respectively. Turkey stands out again, with 14 reversals in four families, viz. Clausiliidae (1), Orculidae (2), Vertiginidae (2) and Enidae (9); the high frequency of sinistrality among the Enidae is conspicuous.

### Chirality Reversals among the Clausiliidae

Keeping in mind the incompleteness of the fossil record and the fact that an even number of repetitive reversals or the extinction rates of dextral and sinistral lineages are hardly recognized, we may conclude that within a period of time of over 65M years, there have been at least 14 and maybe over 20 reversals in chirality among the clausiliid species. Assuming that we are dealing with a stochastic process, this refers to a frequency for such events of once every 4.6–3.2 MY.

### Chirality and Speciation

For the combined recent molluscan faunas of North America, Europe and Turkey not a single sinistral species with a relatively flat shell as defined by Cain [42] is known. Among the faunas examined, only for the Japanese fauna two reversals among broad-shelled taxa may be indicated. Other rare examples of sinistral taxa with flat to globular shells are known, for example, from tropical pulmonate faunas [31] and temperate camaenids, in which four of 18 (22.2%) genera include sinistral species and 34 of 194 (17.5%) species in total are sinistral [43]. Shell slenderness is not a strongly conserved character. ‘Although each superfamily and family can be classified as either mainly tall or flat, both tall and flat species are found in 16 (of 46) families’ [6].

Several authors suggested that the fact that the overwhelming majority of the sinistral gastropod species have shells that are clearly higher than broad might be a consequence of the ease of the initial steps in chiral speciation [12], [19], [22], [33], [40], [44], [45]. For total reproductive isolation to be established, i.e. full speciation, genes acting against interchiral and therefore less successful, copulation attempts should be involved additionally, comparable to what Johnson et al. [29] described for *Partula*. Mirror image mutant snails with globular shells may suffer from strong to maybe complete incompatibility in mating attempts with the majority of wild type individuals [6], [33], [40], [46], so that the initial steps towards single-gene speciation will be most difficult here. In species with slender shells, like for example Clausiliidae and Enidae, interchiral copulation will be more easily achieved, so that in those taxa chiral speciation is less improbable. Difficulties in interchiral mating may cause prompt fixation for reversal, once the reversed morph exceeds 50% in phenotypic frequency under positive frequency-dependent selection. This may explain the relatively frequent chiral speciation in internally fertilizing gastropods, compared to its virtual absence in those animals that reproduce by external fertilization or copulation by genitalia located in the midline and thus experience little interchiral-mating difficulty [5].

In Southeast Asia, sinistral snails survive predation by the snail-eating snakes *Pareas* better than dextrals, because the snakes are specialized to prey on the latter which they more frequently encounter [30]. This predation has accelerated the origin of sinistral species by reversal [31]. Without survival advantages under chirally specialized predation [47], [48] or a reproductive advantage in reducing hybridization [29], however, sinistral snails would probably not differ from dextral ones from the perspective of adaptation to particular niches. Therefore, in a secondary contact between potential sister species evolving in accordance with the single-gene speciation model, resource competition may be severe, resulting in competitive exclusion. Internal selection before hatching may additionally act against the mirror image mutant [5], [49], [50].

In rare cases, chiral speciation may nevertheless result in sympatric and syntopic, congeneric mirror-image species. Uit de Weerd et al. [51] found that all sinistral *Sericata* taxa (Clausiliidae) are vicariant, whereas some of these species occur in sympatry in the same habitat with the more wide-spread *Sericata dextrorsa* (O. Boettger, 1877), the only dextral species in the genus. Apparently, two sinistral species of *Sericata* do not or cannot co-occur, but a sinistral and a dextral one may be found together. Data on niche partition are lacking here. This is to some extent comparable with the occurrence of sinistrality in *Partula* [29].

In North America, *Holospira roemeri* is widely distributed, whereas the other subulinid species have far more restricted ranges [52]. Maybe these contrasting distributional patterns are a consequence of the fact that *H. roemeri* (L. Pfeiffer, 1848) is sinistroid [38], i.e. the final half of its body whorl is curved in such a way that the shell seems to be sinistral at first sight. It is unknown whether that shape has the same consequences for the reproductive behaviour of *Holospira* species as real sinistrality has among other taxa.

The exceptional evolutionary success of the Clausiliidae, according to species numbers, might be a consequence of the clausilial apparatus, without any relation to the unusual, i.e. sinistral, coiling direction of the species in which this structure evolved. A puzzling dimorphism in handedness is known for the clausiliid genus *Alopia* H.&A. Adams, 1855, with a large number of dextral taxa next to many more or less closely related sinistral ones [32]. As long as there is no reliable phylogeny reconstruction for the many so-called species in this genus, it will remain unclear how frequently a polarity shift in chirality has really occurred here. Dextrality is generally accepted as the apomorphic condition for the Clausiliidae. When the dextral species form a monophyletic group, contrary to the opinion of Nordsieck [32], only once a polarity shift occurred in *Alopia*. If not, a hitherto unknown mechanism facilitating reversals may be accepted. Otherwise could be speculated that the ancestral *Alopia* has been dimorphic in chirality. When both morphs dispersed randomly towards the present range of the genus, dextrality or sinistrality may have originated by local positive frequency-dependent selection starting from the various points of departure that were acquired by chance.

For details on the aberrant species of *Amphidromus* with populations of both dextral and sinistral individuals we refer to the literature [2], [12], [24], [25], [26], [27].

### Conclusions

The Clausiliidae is the only speciose family of sinistral gastropods. Where the family is represented, sinistral species may encompass up to about a quarter of the entire molluscan fauna. Where no clausiliids are found, sinistrality is much more rare. As a consequence, the percentages of dextral versus sinistral species may differ conspicuously geographically, depending on the presence or absence of only that family. The presence of other families is less relevant in this respect, but the relatively high percentage of sinistral snail species in Turkey may be connected with the conspicuous, regional radiation of both the Orculidae and Enidae in addition to the Clausiliidae.

Among terrestrial snails, single-gene speciation on the basis of chirality is possible and may have occurred, but as uncommon events. The number of such events resulting in the origin of mirror image species is not closely linked to the percentages of sinistral versus dextral species in a particular region. Shell morphology and the corresponding mating mode, with roughly parallel, slender shells versus opposite, globular shells, are important here. Globular shells and simultaneous, reciprocal mating, and genetic background, i.e. a recessive sinistrality allele, may seriously hamper chiral speciation, while sinistrality may additionally be selected against by internal selection. During the radiation of the Clausiliidae, a family that is characterized by slender shells and dominance of the sinistrality allele (at least in the few cases that have been studied) a successful reversal in coiling direction that left traces in the fossil record or the recent fauna occurred less frequently than once every three to four million years.
